# Intracellular signalling during neutrophil recruitment

**DOI:** 10.1093/cvr/cvv159

**Published:** 2015-05-21

**Authors:** Attila Mócsai, Barbara Walzog, Clifford A. Lowell

**Affiliations:** 1 Department of Physiology, Semmelweis University School of Medicine, Tűzoltó utca 37-47, 1094 Budapest, Hungary; 2 MTA-SE ‘Lendület’ Inflammation Physiology Research Group of the Hungarian Academy of Sciences and the Semmelweis University, 1094 Budapest, Hungary; 3 Department of Cardiovascular Physiology and Pathophysiology, Walter Brendel Centre of Experimental Medicine, Ludwig-Maximilians-University, 80336 Munich, Germany; 4 Department of Laboratory Medicine, University of California, San Francisco, San Francisco, CA 94143, USA

**Keywords:** Leucocytes, Signalling, Neutrophils, Migration, Inflammation

## Abstract

Recruitment of leucocytes such as neutrophils to the extravascular space is a critical step of the inflammation process and plays a major role in the development of various diseases including several cardiovascular diseases. Neutrophils themselves play a very active role in that process by sensing their environment and responding to the extracellular cues by adhesion and de-adhesion, cellular shape changes, chemotactic migration, and other effector functions of cell activation. Those responses are co-ordinated by a number of cell surface receptors and their complex intracellular signal transduction pathways. Here, we review neutrophil signal transduction processes critical for recruitment to the site of inflammation. The two key requirements for neutrophil recruitment are the establishment of appropriate chemoattractant gradients and the intrinsic ability of the cells to migrate along those gradients. We will first discuss signalling steps required for sensing extracellular chemoattractants such as chemokines and lipid mediators and the processes (e.g. PI3-kinase pathways) leading to the translation of extracellular chemoattractant gradients to polarized cellular responses. We will then discuss signal transduction by leucocyte adhesion receptors (e.g. tyrosine kinase pathways) which are critical for adhesion to, and migration through the vessel wall. Finally, additional neutrophil signalling pathways with an indirect effect on the neutrophil recruitment process, e.g. through modulation of the inflammatory environment, will be discussed. Mechanistic understanding of these pathways provide better understanding of the inflammation process and may point to novel therapeutic strategies for controlling excessive inflammation during infection or tissue damage.

## 1. Introduction

Neutrophils play a critical role in host defence against invading pathogens, but they are also critical contributors to tissue damage in immune-mediated disease processes such as autoimmune or autoinflammatory diseases,^1–3^ and they also strongly contribute to various diseases of the cardiovascular system such as atherosclerosis,^4^ myocardial infarction, stroke and ischaemia–reperfusion injury,^5–7^ thrombosis,^8^ as well as small vessel vasculitis.^9^ The contribution of neutrophils to these processes depends on the ability of the cells to traffic between the intravascular and extravascular tissues, for example during the migration to sites of microbial invasion or sterile injury.^10–12^

Neutrophil recruitment from the bloodstream is triggered by an inflammatory tissue microenvironment characterized by accumulation of inflammatory chemokines, cytokines, and lipid mediators, as well as inflammatory changes of the endothelium and, possibly, other subendothelial tissues. The inflammatory microenvironment triggers a multistep process eventually leading to the transmigration of neutrophils through the vessel wall (also called extravasation). Obviously, the accumulation of neutrophils in the extravascular place requires both an inflammatory microenvironment, as well as the intrinsic ability of all cells involved to either conduct (neutrophils) or support (endothelial cells, pericytes) the extravasation process. Understanding of the spatiotemporal control and fine-tuning of the multistep process of the leucocyte (neutrophil) recruitment is critical for our understanding of the overall inflammation process.

There have been a large number of studies dissecting the multiple steps of neutrophil migration across the vessel wall.^10–14^ According to our current understanding, neutrophils first make temporary and reversible interactions with the endothelial surface; this phase is mediated by selectins and is called (fast) rolling. The cells then sense the inflammatory microenvironment within the tissue and microvasculature, which leads to slowing down of the cells (slow rolling), mediated by both selectin and integrin interactions. The cells then begin to firmly adhere to and spread over the endothelium, which is primarily mediated by members of the β_2_-integrin family. Firm adhesion is followed by intraluminal crawling of neutrophils, primarily mediated by the β_2_-integrin Mac-1, which is required for finding the ideal place for transendothelial migration. The next step is the complex process of cell migration through the endothelial layer, either through or between the endothelial cells. On the abluminal side of the endothelium, the basal membrane and the pericites provide additional guidance cues before the leucocytes enter the interstitial space. Additional steps ensure that neutrophils migrate toward the source of inflammatory insult (e.g. microbes) within the extravascular space.

In addition to the leucocyte recruitment cascade, neutrophils also make important contributions to the generation of the inflammatory environment,^15,16^ thus triggering an autoamplification loop and therefore promoting additional leucocyte recruitment. Though this aspect of neutrophil function has received much less attention, recent studies indicate that this may be a very critical contribution of leucocytes to the overall inflammation and leucocyte recruitment process.

Neutrophils use a large number of cell surface receptors to sense the inflammatory microenvironment and direct their cellular responses.^17^ Those include G-protein-coupled chemokine and other chemoattractant receptors, classical immunoreceptors (e.g. Fc-receptors), adhesion receptors such as selecting and integrins, cytokine receptors, as well as a number of cell surface and intracellular pathogen recognition receptors such as Toll-like receptors, NOD-like receptors, and C-type lectins.^17^ All those receptors trigger complex intracellular signalling events leading to various cellular responses including migration through the vessel wall.

Here we review some of the most critical intracellular signalling pathways acting within neutrophils and directing their migration through the vessel wall to the site of infection/inflammation. We will first review chemoattractant receptor signalling and the pathways leading to concomitant polarization of the cells required for directional migration. We will then discuss signal transduction pathways triggered by neutrophil adhesion receptors which are required for the multistep process of navigation within the vessels and across the vessel wall. Finally, we will discuss recent studies revealing important contributions of leucocyte (neutrophil) signalling pathways to the generation of the inflammatory microenvironment with a robust indirect role in the leucocyte recruitment process.

## 2. Signalling by chemoattractant receptors

Neutrophil extravasation and chemotaxis require spatiotemporal regulation of intracellular signalling events. The appropriate subcellular localization of signalling receptors and adhesion molecules along the cellular axis of migration is critical for directing neutrophils out of the vasculature to sites of inflammation. The establishment of this spatiotemporal subcellular organization is referred to as neutrophil polarization. Following attachment and slow rolling of neutrophils along inflamed endothelium, neutrophil polarization is the next step in the leucocyte recruitment cascade. Chemokines and chemoattractants are the primary inflammatory agonists that induce neutrophil polarization and migration.^18^ Hence, the study of the intracellular signalling responses downstream of the chemokine receptors illuminates the mechanisms by which neutrophils establish a polarized state for directed extravasation out of the bloodstream.^19^

### 2.1 G-protein-coupled receptors on neutrophils

Members of the seven transmembrane family of G-protein-coupled receptors (GPCRs) are the primary receptors on neutrophils that mediate chemoattractant signalling and polarization responses. Neutrophils express a large array of these receptors (all of them belonging to the Gi/o-coupled receptor subfamily), allowing them to respond to a tremendous diversity of inflammatory peptides, lipids, and small molecules. The initial intracellular signalling pathways initiated by engagement of GPCRs in neutrophils mimic those pathways described in other cells.^17^ First, ligand binding stabilizes the occupied GPCR in an active signalling conformation, which allows disassociation of Gα and Gβγ subunits from the receptor. The free units diffuse locally within the membrane, acting as second messengers where they regulate the activity of enzymes such as adenylate cyclases, phospholipase C isoforms, phosphatidylinositol (PtdIns) 3-kinases (PI3-kinases or PI3K), as well as ion channels. In neutrophils, the Gβγ subunits play the dominant role in GPCR signalling.^20^ The differential activation of these signalling pathways leads to cell polarization, initiating directed migration (chemotaxis). It is also important to note that many of these agonists and intracellular signalling responses affect other neutrophil functions, such as activation of the NADPH oxidase, release of granules and vesicles containing anti-microbial compounds, and prolongation of neutrophil survival. Generally, GPCR responses are very transient, as another major functional response to activation of these pathways is the endocytic uptake of ligand-bound seven transmembrane receptors, which functions to turn off the signalling pathway.^21^ This process is referred to as receptor desensitization. The process of receptor desensitization is thought to be mediated by receptor phosphorylation followed by binding of arrestin molecules to the ligand occupied receptor, which both facilitates dissociation of the Gα and Gβγ subunits but also targets the receptor for endocytosis.^22,23^ Appropriate receptor desensitization is required for neutrophils to sense chemokine gradients and maintain directional migration.

### 2.2 Neutrophil polarization—how do cells sense gradients?

**Figure 1:**
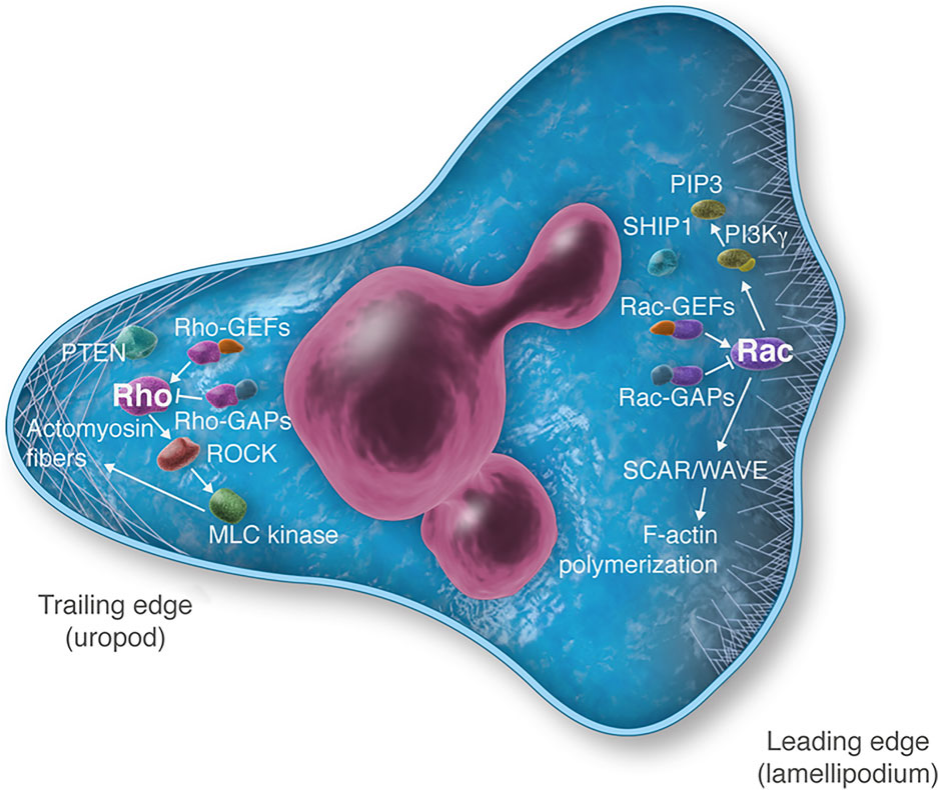
Signalling during gradient sensing and neutrophil polarization. The leading edge (or ‘front’) of the migrating neutrophil is characterized by membrane sheets (lamellipodia) that extent out from the cell body and push the leading edge forward. Lamellopodia are formed by polymerization of F-actin. A number of signalling molecules are enriched in the leading edge that leads to the formation of F-actin. This includes Rac1, Rac2, Rac GEFs (such as DOCK2), Rac GAPs (such as ArhGAP15), PI3Kγ, and PI3Kδ and their products PIP_3_, the SCAR/WAVE complex that directly nucleates the F-actin formation and mAbp, which binds filamentous actin to increase adhesion. The PIP_3_ phosphatase SHIP1, which degrades PIP_3_, is also found at the leading edge. The trailing edge (or ‘back’) of the migrating neutrophil is characterized by the presence of actiomyosin fibres (formed by myosin light chains), which form the uropod, as well as the presence of signalling molecules that induce these fibres. Contraction of actomyosin fibres leads to detachment of the trailing edge from the substratum. The signalling molecules organized in the uropod include Rho GTPases, Rho GEFs (such as Lsc and PDZRho), Rho GAPs (such as ARAP3), and Rho effector molecules such as the ROCK and MLC kinase (which phosphorylate myosin light chains to facilitate their assembly). The PIP_3_ phosphatase PTEN is also found in the rear of migrating neutrophils. Interestingly, most of the β_2_-integrins, which mediate attachment of the neutrophil to the substratum, are located in the rear of the cell. The establishment of the leading vs. trailing edge is self-reinforcing—backness signals inhibit frontness signals and vice versa. Polarization is amplified as cells move up a chemokine gradient, likely through increased signalling at both the front and back of the cell. In contrast, reduction of GPCR signalling, through receptor desensitization, leads to loss of polarization and hence cessation of migration.

A critical step of the neutrophil recruitment process is the translation of extracellular chemokine gradients into an intracellular signalling gradient that directs the migratory machinery of the cells towards the source of the chemoattractant. Upon stimulation with the prototypical neutrophil chemoattractant f-Met-Leu-Phe (fMLF), which binds to the formyl peptide receptors FPR1, FPR2 (also known as ALX), and FPR3, neutrophils undergo a stereotyped progression of steps, over a 2 to 3 min time frame, to rearrange their cytoskeletal structures, change their shape by establishing distinct subcellular structures, and hence become polarized.^24^ With continuous exposure to fMLF, polarization is reversed within ∼10 min.^25^ The readily identifiable domains in the polarized neutrophil include the leading edge (pseudopod), which is characterized by membrane protrusions (lamellipodia, consisting of F-actin bundles that push the cell forward), and a trailing edge (uropod), which contains myosin light chain (MLC2) filaments that facilitate contraction of the rear of the cell (*Figure 1*). The leading edge is established by the action of PI3Kγ, whose products such as PtdIns(3,4,5)P_3_ (PIP_3_) activate Rac GEFs which in turn stimulate Rac GTPases to drive the SCAR/WAVE complex to nucleate F-actin assembly. The trailing domain is enriched in Rho GTPase, which inhibits Rac activity, as well as the presence of the PTEN phosphatase, which degrades PIP_3_. Additionally, Rho action stimulates kinases that act on the myosin molecules. Between these domains is the middle region of the cell, which is characterized by a microtubule lattice that facilitates the dynamic delivery of intracellular molecules to the front and back domains, thus establishing polarity. Ku *et al.*^26^ studied the kinetic crosstalk of intracellular molecules between these domains during fMLF-induced neutrophil polarization, using IF microscopy of cells fixed at multiple time points. This analysis revealed that the initial steps of neutrophil polarization (within 1–5 min of stimulation) occur at the leading edge of the cell, through Rac-mediated actin polymerization, which in turn drives Rho and MLC (and associated MLC kinases) towards the rear of the cell. The overall polarity of the cell is maintained for the subsequent 5–10 min period by flow of molecules from the microtubule domain outward towards both the front and back of the cell. There is a surprisingly dynamic exchange of molecules between these domains, which can be differentially affected by various inhibitors of cytoskeletal rearrangement (such as the F-actin depolymerizer latrunculin or the microtubule stabilizer taxol). The establishment and maintenance of polarization are thought to force directionality on the cell allowing the neutrophil to migrate up the chemokine gradient. In other words, rather than being able to tell the difference between chemoattractant concentrations at the front of the cell vs. the back of the cell, to inform the cell which way to migrate, the establishment of neutrophil polarization forces the cell into a general chemotactic state. As the neutrophil migrates up the chemokine gradient, polarity is enhanced (described by Ku *et al.*^26^ as a feed forward signalling response); hence, the cell moves in the direction of the chemoattactant. Movement away from the chemoattactant source reduces the degree of polarization and the cell stops moving. This self-amplifying loop of increasing chemoattactrant signalling leading to more cell polarization goes on until receptor densensitization occurs. The development of membrane tension may also help sustain neutrophil polarization, by restricting signalling molecules to the leading or trailing edge of the cell.^27^ Interestingly, the GPCRs themselves are evenly distributed over the plasma membrane during chemotaxis.^19^ Thus, the process of neutrophil polarization in response to GPCR signalling likely underlies the ‘chemotactic compass' that allows neutrophils to migrate out of the vasculature and find invading pathogens.

The list of signalling molecules that are differentially localized in polarized neutrophils following GPCR stimulation is large. Besides the PI3Kγ isoform, which is specifically activated by GPCR pathways and is the major producer of PIP_3_ at the leading edge, the 5′ PIP_3_ phosphatase SHIP1 is also found at the leading edge of migrating neutrophils. Indeed, SHIP1 may have a greater regulatory role on neutrophil polarization than the traling edge 3′ PIP_3_ phosphatase PTEN.^28^ Murine neutrophils deficient in SHIP1 manifest increased chemotactic responses both *in vitro* and *in vivo*, whereas PTEN deficient cells migrate normally. Interestingly, GPCR-mediated activation of the NADPH oxidase may also contribute to the feedback loop that amplifies PIP_3_ accumulation in the membrane. Kuiper and colleagues^29^ suggest that NOX2-generated reactive oxygen species may directly down-regulate phosphatases (such as PTEN) to facilitate accumulation of PIP_3_ at the leading edge. Neutrophils lacking NOX2 manifest significant chemotaxis defects *in vitro*. The protein kinases PKCβ and PKD have also been implicated in actin polymerization events downstream of GPCR activation. The PKCβ/PKD signalling molecules may directly alter actin depolymerization at the leading edge of the polymerized neutrophil through phosphorylation of a cofilin phosphatase referred to as SSH2.^30^ In addition, Chen *et al.*^31^ also proposed that ATP released at the leading edge during neutrophil migration generates a secondary gradient that amplifies neutrophil chemotaxis through purinergic receptors.

Similarly, it is not just Rac and Rho GTPases that regulate neutrophil polarity. Among the Rac family members, Rac2 seems to play a dominant role in GPCR-induced chemotaxis, though the relative roles of Rac1 vs. Rac2 may vary depending on chemoattractant type and concentration.^32^ Among the Rac GEFs, which include Vav family members, Tiam1 and DOCK2, suprisingly the unconventional Rac GEF DOCK2 seems to play a dominant role. DOCK2-deficient neutrophils manifest a severe defect in PIP_3_ formation and F-actin polymerization at the leading edge.^33^ Likewise, the Rac GAP ArhGAP15 plays an important role since deficiency of this molecule leads to prolonged Rac activation with increased PIP_3_ accumulation in the lamellipodia.^34^ Other GTPase at the leading edge include the Rap subfamily of GTPases. Deficiency of either Rap1B or its GEF (CalDAG-GEF1) results in poor activation of F-actin formation at the leading lamellipodia.^35^ The Cdc42 GTPase has also been implicated in leading edge PIP_3_ generation. Deficiency of Cdc42, which is localized to the leading edge in polarized neutrophils, also leads to chemotaxis defects.^36^ Interestingly, the Cdc42 effector molecule WASP also contributes to neutrophil polarization, through localization to the trailing edge of migrating neutrophils where it facilitates clustering of β_2_-integrins for adhesion.^37,38^ Besides RhoA, PTEN, and MLC proteins, other trailing edge signalling molecules include the Rho effectors ROCK kinase and mDia proteins that regulate phospho-MLC. Deficiency of ROCK leads to unique tail retraction defect in migrating neutrophils, where the cells leave tiny bits of themselves behind as they push forward.^39^ Similarly, the deficiency of RhoA GEFs (Lsc and PDZRho) leads to alterations in tail end retraction that affect neutrophil chemotaxis (cells tend to form multiple pseudopods and try to migrate in different directions simultaneously).^40,41^ The deficiency of the RhoA GAP ARAP3 also results in altered neutrophil polarization following fMLF stimulation, leading to impaired directional migration.^42,43^ Hence, there are many signalling outputs from the GPCRs that all work together to establish and maintain neutrophil polarity for directed cell migration.

### 2.3 Not all GPCR signalling is the same

The vast majority of *in vitro* studies use formylated bacterial peptides (fMLF), which act through the FPR receptors, as the major agonist. There is an underlying assumption that signalling readouts with this agonist will reflect responses with other GPCR agonists. However, there are clear examples of differential effects of different GPCR agonists on neutrophil polarization and migration, with some agonists (e.g. natural chemokines such as CCL1 or CXCL8) having less profound activities than fMLF in stimulating neutrophil polarization/migration (as well as other functional responses).^44^ Indeed, there is a differential activation of PI3K and downstream MAPKs by these agonists, which suggest both qualitative and quantitative differences in signalling responses by different neutrophil GPCR agonists.^45^ Mechanistically, the different responses of neutrophils to different GPCR agonists may result from different subcellular localization of particular GPCRs. This has been demonstrated in comparison of the FPRs vs. the receptor for lipid agonist platelet activating factor 1 (PAFR).^46^ While neutrophils maintain a large intracellular pool of FPRs (primarily on secretory vesicles that are easily mobilized to the plasma membrane during cell activation), the PAFR is only found on the plasma membrane. Thus, while resting cells will respond relatively equivalently to PAF and fMLF, following priming or cell activation, FPR signalling is dominant. The sequential and hierarchical role of different chemoattractants during neutrophil-mediated inflammation has been convincingly demonstrated in the K/BxN serum-transfer arthritis model.^47,48^ Initial production of LTB_4_, which binds to the BLT1 GPCR on neutrophils, is required for initial recruitment of the first wave of cells into the inflamed joint. Local production of IL-1β by neutrophils leads to release of large amounts of various chemokines (CCL3, CCL4, CCL5, CXCL1, and CXCL2) from tissue resident cells such as synoviocytes, endothelial cells, and macrophages. These chemokines in turn bind to neutrophil CCR1 and CXCR2 to amplify recruitment of cells to the inflamed joint. Sequential action of different GPCR agonists has also been demonstrated in sterile injury models.^49^ Initial neutrophil recruitment is mediated by peptide chemokines such as CXCL2; however, recognition of cellular damage products (mainly from mitochondria) by neutrophil FPR receptors is required for full entry of cells into the inflammatory site.

### 2.4 Other functions of neutrophil chemokine receptors

While the vast majority of studies focus on the roles and mechanisms by which chemokines and GPCR-mediated signalling lead to neutrophil activation (polarization, migration, as well as activation of effector functions), it is also clear that aspects of GPCR signalling work to actively dampen inflammatory responses. The best example of this is the action of ‘pro-resolving’ lipid mediators such as lipoxins or the protein mediators such as annexin A1.^50^ These mediators work to resolve inflammation by limiting neutrophil adhesion and chemotaxis. Many of these mediators tend to be produced during the later stages of inflammatory responses in tissues. While these agents inhibit neutrophil activation and recruitment, they act as strong chemoattractants for monocytes and macrophages (a well as help switch macrophage phenotype to the M2, tissue healing phenotype) which may also promote resolution of acute inflammation.^51,52^ In neutrophils, these pro-resolving mediators signal through the FPR2/ALX GPCR receptor. Amazingly, engagement of the FPR2 receptor by pro-inflammatory agonists (such as the anti-microbial peptide LL-37) leads to activation of the neutrophil polarization and chemotaxis.^53^ The obvious question is how can different ligands engage the same receptor and yet have such differing functional effects? Recent studies suggest that pro-resolving ligands may induce FPR2 homodimerization, leading to p38/MAPK activation, while inflammatory ligands induce FPR2 heterodimerization with other FPR receptors leading to JNK-mediated downstream signalling.^54^ Hence, induction of different receptor conformations may explain how the FPR2 receptor is able to mediate both pro- and anti-inflammatory signals.

In addition to directly activating neutrophil polarization/chemotaxis (as well as other effector functions such as NADPH oxidase assembly), it is also well recognized that GPCR signalling synergizes with other pathways in neutrophils to augment cellular responses. In part, this relates to the idea that GPCR signalling ‘primes' neutrophils for enhanced responses to pathogen molecules. A classic example is the ability of chemoattractant LTB_4_ (which signals through the GPCR BLT1) to enhance signalling through Toll-like receptors (TLR2 and TLR4). Recent studies suggest that these signalling pathways interact at the TAK1 kinase level, which functions to activate NF-κB pathways leading to enhanced cytokine expression by neutrophils that are co-stimulated by LTB_4_ and various TLR ligands.^55^ While direct activation of TAK1 is not a dominant feature of GPCR signalling, it is sufficient to have an additive effect when other pathways (such as TLR signalling) that are well known to proceed through TAK1 are also stimulated in the same cell. It remains to be seen if such receptor signalling ‘crosstalk’ exists between GPCR pathways and other signalling pathways such as tyrosine kinases.

## 3. Signalling by leucocyte adhesion receptors

What are the mechanisms that orchestrate neutrophil recruitment and activation during the acute inflammatory response? In addition to cytokines and other inflammatory mediators, cellular interactions of the neutrophils mediated by adhesion molecules of the β_2_-integrin (CD11/CD18) family play a key role in regulating multiple aspects of this process. They are essential for a variety of cell–cell interactions including slow leucocyte rolling on inflamed endothelial cells, induction of firm adhesion, spreading, intraluminal and abluminal crawling as well as cell–matrix interactions with fibrin, and for example, host–pathogen interactions with complement-opsonized bacteria as well as the activation of various defence functions.^56^ To mediate these complex processes, integrins have to be tightly controlled with respect to their expression, activation, and recycling.^57^ Here, we will discuss the mechanism controlling the functional activation of β_2_-integrins which is of utmost importance for neutrophil trafficking and the fine-tuning of cellular activation in the context of host defence and inflammation.

### 3.1 Neutrophil adhesion receptors of the β_2_-integrin family

Leucocyte adhesion receptors of the β_2_-integrin family (CD11/CD18) are heterodimeric non-covalently bound transmembrane glycoproteins consisting of an α-subunit and a common β-subunit termed CD18. Neutrophils express three different β_2_-integrins, namely LFA-1 (α_L_β_2_; CD11a/CD18), Mac-1 (α_M_β_2_; CD11b/CD18), and gp150/95 (CD11c/CD18). Mac-1, the most abundant integrin on neutrophils, is substantially up-regulated on the cell surface upon neutrophil activation by mobilization from intracellular stores. LFA-1 and gp150/95, the function of which is still enigmatic, are constitutively expressed on neutrophils.^58^ LFA-1 mediates slow leucocyte rolling and induction of firm adhesion by binding to endothelial ICAM-1, whereas Mac-1 plays a predominant role, for example in intraluminal crawling via ICAM-1.^59,60^ ICAM-1-mediated adhesion is also instrumental for neutrophil–pericyte interactions during abluminal crawling, whereas integrins are dispensable for interstitial leucocyte migration but the cells can also switch to integrin-dependent migration modes.^61–64^ At sites of host defence, LFA-1 and Mac-1 are critical for the neutrophils to eventually reach the hotspot of inflammation where the cellular activation takes place in a cluster of neutrophils.^65^ Furthermore, Mac-1 mediates phagocytosis of complement-opsonized bacteria and activates multiple neutrophil functions including the generation of reactive oxygen species (ROS). The fundamental importance of β_2_-integrins for host defence and inflammation becomes evident in patients suffering from leucocyte adhesion deficiency (LAD) type I which lack β_2_-integrin expression due to the absence of the β-subunit CD18 caused by an inherited genetic defect. These patients present with recurrent bacterial and fungal infections characterized by the complete absence of an inflammatory response due to defective neutrophil recruitment and activation.^66,67^ In contrast to LAD type I, LAD type II is caused by impaired selectin function due to defective fucose metabolism, whereas LAD type III is caused by defective β_2_-integrin activation due a genetic defect in kindlin-3 demonstrating that the process of activation is key to β_2_-integrin function.^68–70^

**Figure 2:**
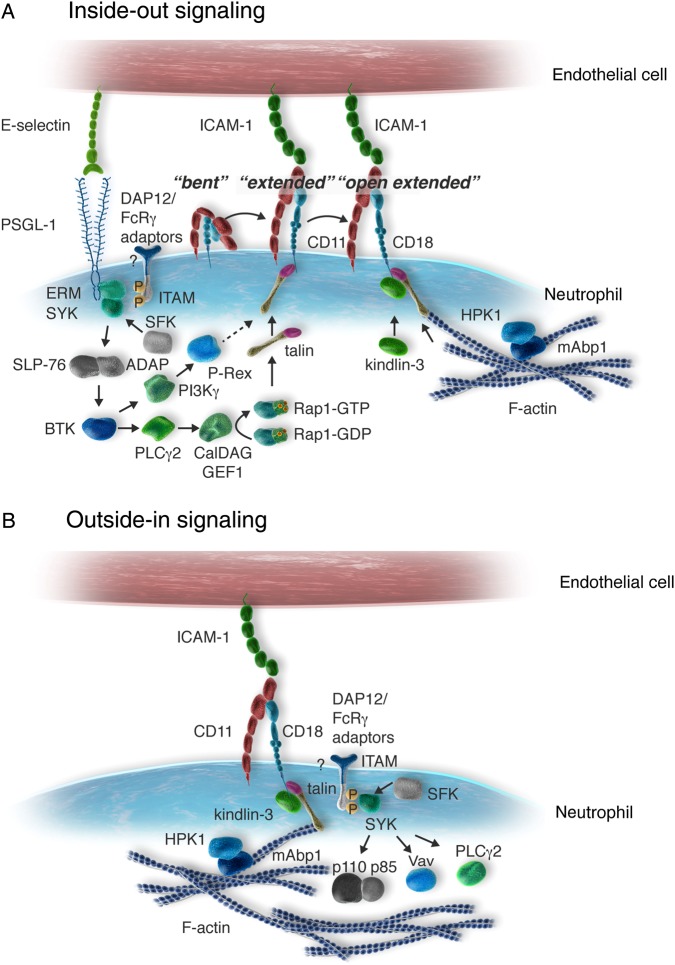
Bidirectional signalling of β_2_-integrins. (*A*) Inside-out signalling of β_2_-integrins induces the shift of the bent conformation with low ligand binding affinity to the extended conformation with intermediate ligand binding affinity and the induction of the open extended conformation with high ligand binding affinity. Signalling is initiated by PSGL-1 upon rolling of neutrophils on E-selectin resulting in the induction of the extended conformation of LFA-1 with intermediate ligand binding affinity that allows slow leucocyte rolling on ICAM-1. During slow leucocyte rolling, neutrophils sense the chemokines presented by the inflamed endothelium via their GPCRs (not shown) which induce signalling events mediating the shift towards the open extended conformation of LFA-1 with high ligand binding affinity and thereby enabling induction of firm arrest. (*B*) Outside-in signalling of β_2_-integrins is initiated upon ligand binding by clusters of high affinity integrins and controls β_2_-integrin-mediated post-adhesion steps including adhesion strenghening, spreading, and intraluminal crawling by fine-tuning the actin cytoskeleton of the migrating neutrophil.

β_2_-Integrins exist in three conformational states characterized by different ligand binding affinities (*Figure 2*). β_2_-Integrins in the bent conformation, which have low ligand binding affinity, are found predominantly on non-activated neutrophils. Upon cellular activation, β_2_-integrins adopt an extended conformation with intermediate binding affinity, a state that is known to be critical for slow leucocyte rolling on ICAM-1. β_2_-Integrins with high ligand binding affinity have an extended, open conformation where the cytoplasmic tails unclasp enabling the association of signalling molecules with the cytoplasmic domains that build the integrin adhesome.^71,72^ The up-regulation of integrin affinity, i.e. integrin activation by intracellular signalling processes, is termed integrin inside-out signalling. In addition to the regulation of ligand binding affinity by inside-out signalling, integrin adhesiveness is controlled by integrin clustering. Integrin clustering occurs upon ligand binding and increases binding avidity. Once the activated integrins bind their ligands, outside-in signalling processes are initiated, orchestrating, for example the fine-tuning of the cytoskeleton required for firm adhesion under flow conditions, spreading as well as intraluminal and abluminal crawling, building of the phagocytic cap, and ROS production.^73^

### 3.2 Signalling by β_2_-integrins during neutrophil recruitment

β_2_-Integrin inside-out signalling can be initiated by GPCRs, selectin ligands, or innate immune receptors during neutrophil rolling on endothelial E- or P-selectin inducing the intermediate affinity state of LFA-1. This conformational shift of LFA-1 allows slow leucocyte rolling on ICAM-1 demonstrating the functional link between signalling via selectin ligands and integrin activation.^74^ The selectin ligands P-selectin glycoprotein ligand 1 (PSGL-1), E-selectin ligand 1 (ESL-1), and CD44 initiate different intracellular signalling pathways in neutrophils. PSGL-1 represents the major selectin ligand on neutrophils and binds P-selectin, E-selectin, and L-selectin. Neutrophil rolling on E-selectin via PSGL-1 activates intracellular signal transduction processes that lead to the induction of the conformational change of LFA-1 as shown in *Figure 2A*. The inside-out signalling cascade elicited by PSGL-1 involves phosphorylation of the ITAM (immunoreceptor tyrosine-based activation motif)-bearing adaptor molecules DAP12 and FcRγ by the Src-family kinases Hck, Fgr, and Lyn.^75^ Together with the ERM protein-mediated association of spleen tyrosine kinase (Syk) with the cytoplasmic tail of PSGL-1,^76^ these steps are crucial for the initiation of inside-out signalling. Syk signals via the adaptor molecules ADAP and SLP-76 resulting in the activation of Bruton's tyrosine kinase (Btk).^77^ Btk in turn activates phospholipase Cγ2 (PLCγ2) and PI3Kγ. Subsequent CalDAG-GEFI and p38 MAPK activation result in activation of the small GTPase Rap1 (25).^78^ Rap1 induces the recruitment of the FERM-domain containing cytoskeletal-associated protein talin to the cytoplasmic tail of the β-subunit of LFA-1. Talin links the integrin to the cytoskeleton and allows its conformational shift to the intermediate affinity state that mediates slow rolling on ICAM-1.^79^ In addition, the Rac-specific gunanine nucleotide exchange factor P-Rex1 is critically involved in regulating the intermediate affinity of LFA-1.^80^ In contrast to slow leucocyte rolling, induction of firm adhesion under flow conditions requires the induction of the high affinity conformation of LFA-1 which depends on GPCR signalling and is mediated by kindlin-3.^81^ In addition to talin-1, kindlin-3 binds to the cytoplasmic domain of the β-subunit of LFA-1, thereby stabilizing its open extended conformation under physiological flow conditions *in vivo* which is important for firm neutrophil arrest on ICAM-1.^82^ Moreover, the mammalian actin binding protein 1 (mAbp1) is critically involved in the induction and/or stabilization of the high affinity conformation of LFA-1.^83^ In contrast to Kindlin-3, mAbp1 is only required for firm adhesion under flow conditions when high shear forces are applied to the integrin, whereas mAbp1 is completely dispensable for adhesion under static conditions. The same holds true for the haematopoietic progenitor kinase 1 (HPK1) which is constitutively associated with mAbp1 in neutrophils and is critically required for the induction of the high affinity conformation of LFA-1 and subsequent firm neutrophil adhesion to ICAM-1 under flow conditions.^84^

In addition to PSGL-1, the E-selectin ligand ESL-1 mediates neutrophil rolling.^85^ Although E-selectin binding is significantly reduced in the absence of ESL-1, neutrophil recruitment namely initial rolling, induction of adhesion, and extravasation are unaffected in ESL-deficient neutrophils under diverse inflammatory conditions *in vivo* indicating a functional compensation by PSGL-1. However, neutrophil trafficking is severely compromised in PSGL-1 and ESL-1 double deficient neutrophils compared with PSGL-1 knockout neutrophils indicating an important functional cooperation between ESL-1 and PSGL-1. In contrast to mature neutrophils, ESL-1 is fundamentally important for homing of haematopoietic progenitor cells (HPCs) to the bone marrow indicating its differential role in various leucocyte subsets.^86^ Together these results suggest that ESL-1 is dispensable in neutrophils for transducing intracellular signals leading to the conformational change of LFA-1 into the intermediate affinity state allowing slow neutrophil rolling on ICAM-1. In contrast, ESL-1 signalling in neutrophils induces activated Mac-1 clusters at the leading edge of crawling neutrophils. In addition to affinity regulation, this process is a key to the regulation of integrin adhesiveness. This activation step allows, for example the interaction between neutrophils and red blood cells in a humanized mouse model of sickle cell disease resulting in lethal vascular occlusion. In a model of antibody-induced acute lung injury, the same mechanism allows the interaction between neutrophils and platelets resulting in ROS production by neutrophils that causes vascular leakage and organ injury. Here, inhibition of Src-family kinases but not Syk shows protective effects.^87^ In contrast to PSGL-1 and ESL-1, the role of CD44 seems to be mainly restricted to neutrophil sequestration in liver sinusoids.^88^ In addition to its role in inside-out signalling, selectin ligands, and especially CD44, are also involved in the induction of adhesion molecule clustering which is critical for the control of integrin avidity.^85,89^

Besides selecting ligands and GPCR-based mechanisms, recent studies have also shown activation of β_2_-integrins and initiation of slow neutrophil rolling upon ligation of innate immune receptors such as TLR2, TLR4, and TLR5. Similar to the above pathways, this TLR-induced inside-out activation of β_2_-integrins is also mediated by the Rap1 GTPase and maybe involved in triggering neutrophil adhesion and extravasation either directly through microbial pathogens^90,91^ or by an autocrine mechanism through MRP8/14 molecules released from activated neutrophils.^91^

Outside-in signalling via high affinity β_2_-integrins occurs upon ligand binding, for example to endothelial ICAM-1 and depends on integrin clustering which increases integrin avidity.^92^ Kindlin-3 and LFA-1 co-localize with the membrane store-operated Ca^2+^ channel Orai1 that controls Ca^2+^ signalling and thereby promotes integrin clustering.^93^ During neutrophil trafficking, outside-in signalling processes are important for different β_2_-integrin-dependent post-adhesion functions including adhesion strengthening, spreading, and intraluminal crawling.^94,95^ In analogy to the intracellular signalling cascade initiated by PSGL-1, Src-family kinases phosphorylate the ITAM-bearing transmembrane adapters DAP12 and the Fc receptor γ-chain leading to the translocation of Syk to the lamellipodium of adherent neutrophils and its activation, as shown in *Figure 2B*.^96–98^ Signalling proceeds via interaction of Syk with different downstream molecules, e.g. PLCγ2, the guanine nucleotide exchange factor Vav1, the p85 regulatory subunit of class IA PI3-kinases, and mAbp1.^99–103^ The functional impact of Syk for β_2_-integrin-dependent sustained adhesion and post-adhesion functions *in vivo* becomes evident in wild-type mice reconstituted with a Syk-deficient haematopoietic system. Here, the absence of Syk severely compromises sustained neutrophil adhesion, adhesion strengthening, spreading, and extravasation.^96,97^ Therefore, Syk appears to be important for neutrophil recruitment in models of acute inflammation where neutrophil trafficking is directly studied by intravital microscopy of the inflamed cremaster muscle in mice, for example upon superfusion with the bacteria-derived tripeptide fMLF within minutes after the onset of the experiment.^104,105^ Within neutrophils, Syk co-localizes with Vav1, which mediates intraluminal crawling.^106,107^ Cells expressing a Syk mutant that lacks the Vav binding site generate multiple lamellipodia upon activation and show migration defects revealing the functional role of the interaction between Syk and Vav for neutrophil trafficking.^104^ In addition, Syk also interacts with the regulatory subunit p85 of PI3Kδ leading to the enrichment of PI3Kδ at the leading edge.^103,108^ This translocation results in the activation of PI3K and the generation of PIP_3_ which is important to maintain cell polarity during neutrophil chemotaxis.^109^ Furthermore, the stabilization of the high affinity conformation of the β_2_-integrins under flow conditions critically depends on mAbp1.^83^ In addition to induction of firm adhesion, mAbp1-deficient neutrophils are compromised in β_2_-integrin-dependent post-adhesion functions namely adhesion strengthening, spreading, and intraluminal crawling under flow conditions.^83^ Similar effects are observed in the absence of HPK1, a known mAbp1-interacting protein.^84,110^ HPK1 co-localizes with mAbp1 and actin at the leading edge of polarized neutrophils. It plays not only an important role in the induction of adhesion as mentioned above but also in adhesion strengthening, spreading, and intraluminal crawling.^84^ mAbp1 and HPK1 are also critical for inside-out signalling of the integrin allowing the induction of the high affinity state as mentioned above. Given that this step may result from a shift of the intermediate affinity state where the ligand is already bound transiently, integrin signalling via mAbp1 and HPK1 may already be bidirectional at this point.

It is also important to know that signalling via leucocyte adhesion receptors alone is only able to induce transient neutrophil–endothelial cell interactions in most models. Sustained neutrophil adhesion to the inflamed endothelium as well as efficient extravasation depends on soluble inflammatory mediators including, for example cytokines or lipid mediators. These mediators can very efficiently induce the activation of β_2_-integrins by signal transduction pathways which are independent of the upstream signalling effectors employed by adhesion molecules (such as Syk^111^) but involve the same downstream effectors like Rap1, Talin, and Kindlin-3 as outlined elsewhere.^14,112^

## 4. Indirect effects of leucocyte signalling on inflammatory cell recruitment

While most studies aiming to understand the role of leucocyte signalling in leucocyte recruitment focused on intracellular pathways directly involved in cellular changes during the migration/extravasation process, a number of recent studies indicate that leucocyte signalling may also contribute to the development of the inflammatory environment and therefore indirectly affect leucocyte migration.

In this section, we will discuss such positive feedback processes triggering leucocyte migration to the site of inflammation. We will only focus on situations where activation of a given cell type indirectly triggers recruitment of the same cell type. Situations where activation of one leucocyte lineage triggers activation of another lineage will not be discussed because of the large number of such examples and also because that issue is more relevant to the overall orchestration of the immune/inflammatory response rather than the mechanism of leucocyte recruitment.

### 4.1 The role of leucocytes in generating a chemoattractant inflammatory environment

It is well known that leucocyte activation triggers the release of various pro-inflammatory agents. However, the fact that the release of those agents may positively feed back to the migration of the same lineage, and is therefore critical to leucocyte recruitment, is much less appreciated.

A positive feedback effect of leucocyte-derived chemoattractants is best exemplified by the effect of neutrophil-derived LTB_4_ on other neutrophils. A seminal early paper showed that leucotriene B molecules released from activated neutrophils trigger activation and chemokinetic migration of other neutrophils.^113^ The role of neutrophil-derived LTB_4_ was also shown to be critical for the propagation of inflammation in K/BxN serum-transfer arthritis, a neutrophil-dependent *in vivo* model of arthritis.^47,114^ Genetic deficiency of the enzymes responsible for LTB_4_ synthesis (5-lipoxygenase or LTA_4_ hydrolase) or the LTB_4_ receptor BLT1 protected mice from arthritis development in this model.^47,114^ Interestingly, adoptive transfer of wild-type neutrophils to 5-lipoxygenase-deficient mice restored arthritis development and the accumulation of neutrophils (including 5-lipoxygenase-deficient recipient cells) at the site of inflammation^114^ indicating an important role for neutrophil-derived LTB_4_ in feedback amplification of neutrophil recruitment. This was in agreement with the accumulation of LTB_4_ at the site of inflammation *in vivo* and the parallel release of LTB4 from neutrophils but not macrophages upon immune complex (IC)-induced *in vitro* activation in our own hands.^115^

More recent studies provided further evidence for LTB_4_-mediated autoamplification of neutrophil recruitment. In an *in vitro* study, neutrophil-derived LTB_4_ promoted the migration of neutrophils towards formyl peptides.^116^ In mixed neutrophil preparations, wild-type neutrophils were able to promote the directed migration of FPR1-deficient neutrophils (which are not sensitive to formyl peptide stimulation) towards the chemoattractant source. Importantly, 5-lipoxigenase-deficient neutrophils were not able to trigger the migration of FPR1-deficient neutrophils,^116^ indicating that neutrophil-derived LTB_4_ generates a secondary gradient which triggers the migration of neutrophils towards the source of the primary (e.g. formyl peptide) gradient. This is also in agreement with the amplification of IC-induced neutrophil activation by autocrine release of neutrophil-derived LTB_4_.^117^

Several further recent studies provided additional *in vivo* evidence for LTB_4_-mediated amplification of neutrophil recruitment. Neutrophil-derived LTB_4_ was critical for the recruitment of further neutrophils to the site of mechanical injury and for the immunization against epicutaneous ovalbumin in a mouse model of atopic dermatitis.^118^ Furthermore, laser-induced tissue injury has recently been shown to trigger interstitial migration of neutrophils from a longer distance towards the site of tissue damage.^65^ This accumulation (also called a ‘swarming behaviour’) required both 5-lipoxigenase and BLT1, and further studies revealed that neutrophil-derived LTB_4_ was required as an amplifier of the neutrophil recruitment process.^65^

**Figure 3:**
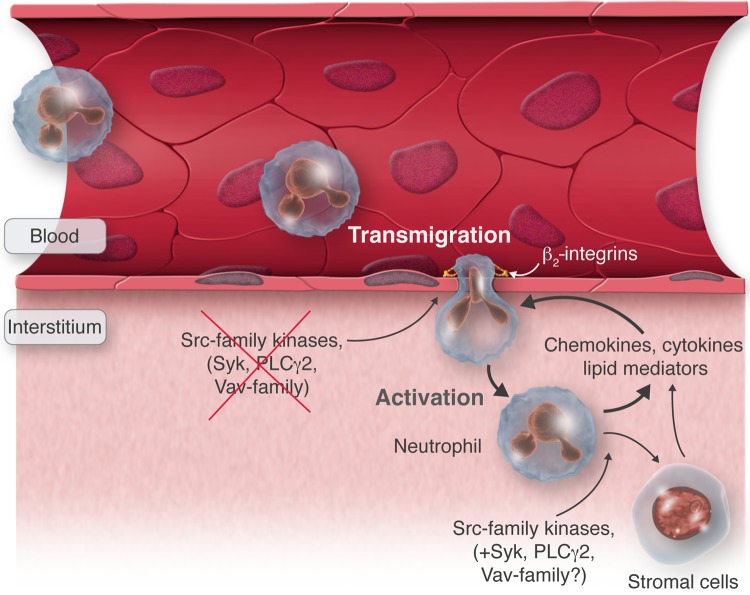
Indirect effect of neutrophil signalling on leucocyte recruitment. At the site of inflammation, neutrophils migrate through the endothelium to the interstitium where they release pro-inflammatory mediators triggering, either directly or through activation of stromal cells, the recruitment of additional neutrophils. β_2_-integrins are required for the intrinsic capacity of neutrophils to migrate through the vessel wall, whereas Src-family kinases (and likely Syk, PLCγ2 and Vav family members) are critical for the release of pro-inflammatory mediators and hence the generation of the inflammatory environment.

Besides amplification through LTB_4_, there are a number of other inflammatory mediators released by neutrophils that promote the recruitment of other neutrophils in an autoamplifying fashion. Neutrophils are rich sources of chemokines,^15^ many of which (e.g. CXCL1, CXCL2, CXCL8) act on neutrophils themselves. Adoptive transfer of wild-type neutrophils to BLT1-deficient mice was able to restore arthritis development in the K/BxN serum-transfer model, and it also triggered recruitment of BLT1-deficient recipient neutrophils.^47^ Besides LTB_4_, IC activation of neutrophils also triggered the release of neutrophil-acting chemokines (e.g. CXCL2) that are also present at the site of autoantibody-induced *in vivo* inflammation.^115^ Therefore, neutrophils appear to release neutrophil-attracting inflammatory mediators other than LTB_4_. A further follow-up study showed that an initial wave of LTB_4_-driven neutrophil recruitment is followed by additional waves triggered by chemokine release at the site of inflammation.^48^ Interestingly, this second wave is induced by neutrophils previously recruited to the site of inflammation, either directly by release of the neutrophil-active chemokine CXCL2 from neutrophils themselves or by the release of IL-1β from the recruited neutrophils which triggers release of neutrophil-active chemokines such as CXCL1, CXCL5, or CCL9 from stromal cells and synovial macrophages.^48^ Therefore, neutrophils amplify their own recruitment in an autocrine manner using a lipid-cytokine-chemokine cascade during autoantibody-induced arthritis (*Figure 3*). The activation of this cascade further requires additional signals mediated by C5a and Fcγ receptors.^119^

Most of the above discussion relates to amplification loops involved in recruitment of neutrophils to the site of inflammation. However, similar amplification loops may also be functional within other leucocyte lineages. In addition to neutrophil-derived neutrophil-attracting chemoattractants, we have also observed dramatic accumulation of chemokines acting on monocyte recruitment (e.g. CCL2 and CCL3) at the site of inflammation during K/BxN serum-transfer arthritis *in vivo* and we found that those chemokines can be released from IC-stimulated macrophages *in vitro*.^115^ Those results suggest that macrophages at the site of inflammation trigger the recruitment of their own precursors (monocytes) from the bloodstream by releasing monocyte-active chemokines to the extracellular space.

### 4.2 Leucocyte signalling in the generation of the inflammatory environment

The existence of the above autoamplification loop raises the possibility that leucocyte signalling pathways contribute to leucocyte recruitment not (or not only) by being directly involved in the migration process but (also) by being required for the release of autocrine mediators recruiting additional cells of the same lineage.

Tyrosine kinase pathways activated by leucocyte immunoreceptors (B- and T-cell receptors and Fc-receptors) and adhesion receptors (integrins and, to some extent, selectin ligands) are mediated by Src-family kinases, as well as members of the Syk/ZAP-70 tyrosine kinase, the PLCγ phospholipase and the Vav guanine nucleodite exchange factor families. We and others have shown that mice lacking the myeloid Src-family kinases Hck, Fgr, and Lyn,^115^ the Syk tyrosine kinase,^120,121^ the PLCγ2 phospholipase^99,122^ or the Vav1, Vav2, and Vav3 exhange factors^122^ are completely protected from arthritis development in the K/BxN serum-transfer model. Whenever tested, deletion of the signalling proteins from the haematopoietic compartment was sufficient to abolish arthritis development,^99,115,120,121^ and in the case of Syk, it has even been shown to be due to Syk deletion in the neutrophil compartment.^121^ Importantly, the Hck^–/–^Fgr^–/–^Lyn^–/–^, Syk^–/–^, PLCγ2^–/–^, and Vav1^–/–^Vav2^–/–^Vav3^–/–^ mutations all caused complete defects in leucocyte recruitment to the site of inflammation^99,115,120,122^ and the overall phenotype strongly resembled that of CD18^–/–^ mice lacking all β_2_-integrins.^123,124^

CD18^–/–^ mice show a cell-autonomous defect of migration of leucocytes to the site of inflammation.^96,115^ The similarity of the Hck^–/–^Fgr^–/–^Lyn^–/–^, Syk^–/–^, PLCγ2^–/–^, and Vav1^–/–^Vav2^–/–^Vav3^–/–^ phenotypes to that of CD18^–/–^ mice, together with the known role of Src-family kinases, Syk, PLCγ2, and Vav family members in integrin outside-in signal transduction^96,99,106,125–127^ suggested that the protection of all those mutants from leucocyte accumulation and arthritis development are due to a cell-autonomous defect of CD18-mediated leucocyte migration. However, several lines of evidence suggest that this is not the case. Neutrophils from all of the above mutants migrated normally in *in vitro* Transwell assays,^96,99,115,127^ and Vav1^–/–^Vav3^–/–^ neutrophils showed normal two-dimensional migration despite reduced adhesion *in vitro*.^106^ No defects of the accumulation of Hck^–/–^Fgr^–/–^Lyn^–/–^, PLCγ2^–/–^, or Vav1^–/–^Vav2^–/–^Vav3^–/–^ neutrophils were observed in thioglycollate-induced sterile peritonitis.^122,128^ In addition, mixed bone marrow chimeric experiments, which allowed the parallel analysis of the migration of wild-type and knockout cells within the same animals, failed to reveal any cell-autonomous requirement for Src-family kinases, Syk, or PLCγ2 in the migration of neutrophils to the site of inflammation in autoantibody-induced arthritis^115,121^ or thiogllycollate-induced peritonitis.^96,99^ In many of those cases, the experiments were performed with parallel assays on CD18^–/–^ neutrophils which consistently showed a dramatic cell-autonomous neutrophil migration defect.^96,115^ Other unrelated approaches such as *in vivo* cremaster muscle migration assays,^99^ thrombohaemorrhagic vasculopathy,^129^ or zebrafish studies^130^ also failed to reveal substantial defects in neutrophil migration upon deficiency of the above signalling molecules or revealed moderate recruitment defects in Syk^–/–^ mutants^104^ which cannot account for the complete protection from disease development in autoantibody-induced arthritis.^99^

An alternative explanation for the defective accumulation of the above mutant neutrophils at the site of inflammation is defective release of neutrophil-derived pro-inflammatory mediators, leading to defective generation of an inflammatory, chemoattractant environment. This has been confirmed in detail in case of the Hck^–/–^Fgr^–/–^Lyn^–/–^ mutants.^115^ Hck^–/–^Fgr^–/–^Lyn^–/–^ mice showed not only lack of infiltrating neutrophils but also complete absence of chemokines, cytokines, and lipid mediators (including IL-1β, CCL3, CXCL2, and LTB_4_) in the synovial tissue in K/BxN serum-transfer arthritis.^115^ Importantly, Hck^–/–^Fgr^–/–^Lyn^–/–^ neutrophils failed to release chemokines, cytokines, and lipid mediators (including IL-1β, CCL3, CXCL2, and LTB_4_) when stimulated by immobilized ICs *in vitro*,^115^ suggesting that the primary defect is in the release of inflammatory mediators from leucocytes. Similarly, Elliott *et al.*^121^ tested neutrophils from the synovial tissue of mixed chimeric mice having both wild-type and Syk-deficient neutrophils and subjected to K/BxN serum-transfer arthritis. They observed that Syk-deficient neutrophils were unable to produce TNF-α upon *ex vivo* restimulation with ICs or LPS,^121^ indicating a critical role for Syk in pro-inflammatory cytokine release of neutrophils. In addition, arthritic treatment-induced up-regulation of IL-1, IL-6, and TNF-α in the synovial tissue extract was abrogated both by the PLCγ2^–/–^ and by the Vav1^–/–^Vav2^–/–^Vav3^–/–^ mutations.^122^

The above experiments mostly focused on autoantibody-induced arthritis models. However, Hck^–/–^Fgr^–/–^ mice were also protected from LPS-induced early acute lung injury, in particular from transmigration of neutrophils from the blood to the bronchoalveolar lavage fluid (G. Berton, personal communication). This was accompanied by reduced chemokine levels in the lung *in vivo* and by reduced *in vitro* generation of chemokines and cytokines by LPS-stimulated neutrophils. Importantly, intratracheal instillation of CXCL2 was able to restore accumulation of neutrophils in the bronchoalveolar lavage fluid even in Hck^–/–^Fgr^–/–^ mice (G. Berton, personal communication), indicating that Hck^–/–^Fgr^–/–^ neutrophils are intrinsically able to migrate through the bronchial wall if an appropriate chemotactic gradient is present.

Besides amplification of neutrophil recruitment, intracellular signalling pathways may also be involved in promoting the recruitment of other leucocyte lineages. As an example, not only neutrophils and neutrophil-attracting chemokines, but also macrophages and monocyte-attracting chemokines (such as CCL2 and CCL3) showed defective accumulation at the site of autoantibody-induced inflammation in Hck^–/–^Fgr^–/–^Lyn^–/–^ mice.^115^ IC-induced *in vitro* release of CCL2 and CCL3 was also strongly reduced in Hck^–/–^Fgr^–/–^Lyn^–/–^ macrophages.^115^ Those results suggest that macrophages at the site of inflammation trigger the recruitment of their own precursors (monocytes) from the bloodstream through a mechanism requiring Src-family tyrosine kinases.

An obvious question is which cell surface receptors utilize the above tyrosine kinase pathway to trigger the release of pro-inflammatory mediators. Though GPCR receptors may utilize Src-family kinases,^126,131^ they do not appear to be the predominant GPCR signal transduction molecules^17^ and Syk is not involved in GPCR signal transduction either.^111^ Though several studies suggest that Src-family kinases and Syk are critical for β_2_-integrin-mediated neutrophil activation,^96,125,126^ the fact that neutrophil activation is mediated by Mac-1 whereas K/BxN serum-transfer arthritis requires LFA-1^115,123^ suggests that this is not the case. Additional studies of IC-induced neutrophil activation suggest that the role of Src-family kinases (and, likely, Syk, and PLCγ2) in arthritis development is likely due to their role in Fc-receptor-induced release of pro-inflammatory mediators from neutrophils.^99,115^

Taken together, signalling pathways mediated by Src-family kinases and other tyrosine phosphorylation pathway components are critically involved in the *in vivo* migration of myeloid leucocytes to the site of inflammation, but this appears not (or not only) to be due to an intrinsic requirement for these molecules in neutrophil/monocyte migration but (also) to their role in the leucocyte-mediated generation of an appropriate chemoattractive inflammatory environment (*Figure 3*). In general, neutrophils have the ability to translate even weak environmental cues into site-directed trafficking profiles especially by signalling via leucocyte adhesion receptors sensing the non-soluble inflamed microenvironment which may be critical for the early onset of the initial phase of an immune response when only a very few bacteria have infiltrated the tissue. Once neutrophil-driven amplification loops initiate and perpetuate a robust inflammatory response, additional mechanism take over and orchestrate the immune response.

## 5. Relevance to cardiovascular diseases

Recent studies have indicated that neutrophils contribute to diverse cardiovascular diseases ranging from atherosclerosis through ischaemic heart and brain diseases to thrombosis and vasculitis.^1,4–9^ The signalling processes discussed in this paper likely contribute to the development and progression of those diseases, and they may even serve as potential targets of therapeutic intervention. As recent examples, annexin A1 and its receptor FPR2 have been shown to be negative regulators of chemokine-induced integrin activation and atherosclerotic lesion formation,^132^ and components of integrin signal transduction pathways have been proposed to be critically involved in ischaemia–reperfusion injury in the kidney.^77^ These and other studies highlight the importance of leucocyte signal transduction in cardiovascular disease processes.

## 6. Concluding remarks and future directions

Studies during the last several years have made it very obvious that intracellular signal transduction in leucocytes make a major contribution to the recruitment of these cells to the site of infection and inflammation, and it is therefore a critical component of the inflammation process. Though we have began to understand how neutrophil signalling contributes to sensing of the chemoattractant gradient, activation of adhesion receptor function, and the various routes of amplification of the recruitment process, a large number of questions remain to be resolved.

It is widely believed that the different inflammatory disease processes, the different vascular beds of the various organs, and the different routes of triggering neutrophil extravasation utilize very different signalling pathways within the cells. Therefore, it may be naive to seek a single unified concept of neutrophil recruitment, and future experiments may indeed reveal a diverse signalling armamentarium used by neutrophils (and other leucocytes) under different inflammatory conditions.

There are also apparent discrepancies between acute and chronic inflammation models, as exemplified by the apparent cell-autonomous role of tyrosine kinase signalling pathways in acute but not in more chronic inflammation disease models (such pathways apparently play non-cell-autonomous roles in chronic inflammation). This may be due to the generation of robust inflammatory stimuli that can overcome or bypass the requirement of signalling processes induced by leucocyte adhesion receptors or activation of redundant intracellular signalling pathways, for example driven by the co-evolution of pathogens and the mammalian immune system.

A more specific unresolved question is the involvement of outside-in signalling of neutrophil integrins during migration. While a large number of studies suggest that integrin outside-in signalling is critical for several steps of the neutrophil recruitment process (firm adhesion, spreading, intravascular crawling), the apparent ability of neutrophils deficient in key integrin outside-in signalling components to migrate along chemotactic gradients *in vitro* and *in vivo* raises the possibility that integrin outside-in signalling is less critical for the overall migration process.

It is also mostly unclear how the predominantly animal and *in vitro* experiments described here can be translated to better understanding and therapy of human inflammatory diseases. The efforts of developing tyrosine kinase inhibitors for the therapy of inflammatory diseases^133^ nevertheless promises a translational potential of the experiments described in this review and suggests that the efforts aimed at understanding the intricate molecular details of leucocyte recruitment may provide substantial benefit to our society.

## Funding

Work in the authors' laboratories was supported by the European Union's FP7 Cooperation Program (TARKINAID project No. EU FP7 to A.M. and B.W.), the Lendület program of the Hungarian Academy of Sciences (LP2013-66 to A.M.), the Deutsche Forschungsgemeinschaft (SFB 914/TP A02 to B.W.), and the NIH (RO1AI65495, RO1AI68150, and RO1AI113272 to C.A.L.). A.M. was a recipient of a Wellcome Trust International Senior Research Fellowship (Grant No. 087782). Funding to pay the Open Access publication charges for this article was provided by the Wellcome Trust.
